# Investigation of Impacts on Printed Circuit Board Laminated Composites Caused by Surface Finish Application

**DOI:** 10.3390/polym13193203

**Published:** 2021-09-22

**Authors:** Denis Froš, Karel Dušek, Petr Veselý

**Affiliations:** Department of Electrotechnology, Faculty of Electrical Engineering, Czech Technical University in Prague, Technická 2, 166 27 Prague 6, Czech Republic; dusekk1@fel.cvut.cz (K.D.); veselp13@fel.cvut.cz (P.V.)

**Keywords:** resin, glass cloth, substrate, glass transition temperature, surface finish, pad cratering, thermal resistance

## Abstract

The purpose of this study was to compare the strength of the bond between resin and glass cloth for various composites (laminates) and its dependence on utilized soldering pad surface finishes. Moreover, the impact of surface finish application on the thermomechanical properties of the composites was evaluated. Three different laminates with various thermal endurances were included in the study. Soldering pads were covered with OSP and HASL surface finishes. The strength of the cohesion of the resin upper layer was examined utilizing a newly established method designed for pulling tests. Experiments studying the bond strength were performed at a selection of laminate temperatures. Changes in thermomechanical behavior were observed by thermomechanical and dynamic mechanical analyses. The results confirmed the influence of the type of laminate and used surface finish on bond strength. In particular, permanent polymer degradation caused by thermal shock during HASL application was observed in the least thermally resistant laminate. A response to thermal shock was detected in thermomechanical properties of other laminates as well, but it does not seem to be permanent.

## 1. Introduction

The interconnection of electrical components through printed circuit boards (PCBs) has been conducted for several decades. In terms of continual development in the electronics industry that allows placing electronic devices in areas with demanding conditions, the properties of the materials used for PCB production must correlate with the requirements of those devices. Therefore, the evaluation of the properties of commonly utilized materials is important for confirming the compatibility for operation in a supposed environment. If ordinary materials are not suitable, materials with enhanced parameters must be selected for manufacturing. However, testing of materials with better properties should be carried out to assess deployment with existing technology and other materials involved in PCBs. Laminates for PCB manufacturing are composites, i.e., it is a system of filler (reinforcement) and resin. A significant property to be tested is the temperature endurance of the composite (usually called a substrate in the PCB industry) that is dominantly determined by the resin. PCBs can be affected by high temperature during the soldering process and, subsequently, by operating conditions that the device is subjected to. Unresolved thermal compatibility may lead to a failure directly on the PCB and to a subsequent dysfunction of the complete device.

Resins that are used for impregnating the filler consist of amorphous polymers. For these polymers, glass transition temperature (Tg) is defined. Under Tg, the polymer is in solid-state, but short-range interconnections break up by heating the material closer to Tg temperature. Molecules of polymer with temperatures over Tg receive enough energy to move freely around. It results in a rubbery state of the material including a change in the physical properties such as the coefficient of thermal expansion (CTE), heat capacity, and mechanical properties [1,2]. CTE changes in the direction of the *z*-axis caused by crossing Tg must be carefully monitored, because multiple soldering or thermal cycles with an upper-temperature limit above the Tg raises the possibility of damaging the plated barrels and vias [3]. Cracking is caused by considerable differences in the CTEs of copper and the substrate in the vertical direction.

Epoxy resins are compounds comprising two or more epoxy groups. These groups react with a wide range of curing agents [4]. Another aspect governing the Tg is the number of epoxide groups in a polymer structure. In addition, additives, the rate of cross-linking determined by the curing agent, and the curing conversion impact on the Tg value as well as other properties and the resin’s performance [5,6,7]. Higher cross-link density is responsible for an increase in temperature, chemical, and moisture resistance. On the other hand, it also results in a less flexible and more brittle material. A typical range of Tg that can be achieved for epoxy resin is 135–185 °C. Ehrler [8] compared the properties of two curing agents used in FR4 laminates. The laminate with a phenol novolac curing system is more suitable for higher thermal requirements than the dicyandiamide (DICY) hardener. Another resin must be chosen in case of specialized higher temperature applications. For example, bismaleimide triazine or polyimide resin produces high-Tg laminates. The possibility of a fall in Tg as a consequence of thermal degradation or moisture absorption must be taken into account, too [9,10]. Applicable techniques for experimental Tg determination are thermomechanical analysis (TMA), dynamic mechanical analysis (DMA), or differential scanning calorimetry (DSC). The two former methods were also used in this study.

Warpage is an effect occurring when the PCB is exposed to thermal stress. Current PCBs in mobile devices with complicated designs are prone to this adverse behavior. Xia et al. [11] experimentally proved that twisting or bowing is more significant for thinner PCBs. Warpage may be further aggravated by the release of residual stress induced in the substrate during the lamination process [12]. Several other studies [13,14] focused on modeling this issue in order to predict the warpage of PCBs. It helps to avoid connection failure following re-design of a product. Moreover, in the case of a relatively larger component with more soldered connections (e.g., ball grid array) that undergoes warpage itself due to the fact of CTE mismatch of the component, mechanically weak solder joints (called head-in-pillow) may occur [15]. Tearing off of the soldering pad from the substrate may appear during soldering while the joint has already solidified and warping of the assembly is still ongoing or at field conditions when CTE differences in the substrate and mounted components raise the stress concentration [16,17]. This failure is known under the term pad cratering.

**Figure 1:**
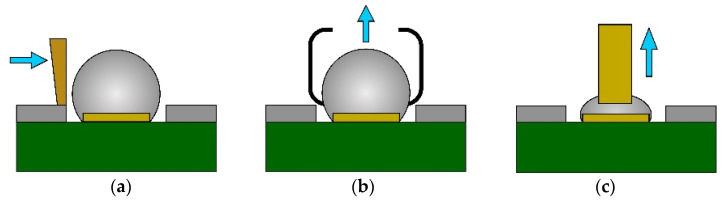
Methods for pad cratering evaluation: (**a**) ball shear; (**b**) ball pull; (**c**) pin pull.

Pad cratering evaluation has been a subject of many studies. A consequence of stress concentration is crack initiation. Its propagation continues at the glass cloth and resin interface, resulting in a separation of the copper pad out of the substrate. It is a critical failure that cannot be reworked. Roggeman et al. [18] investigated the dependency of pad cratering on filled and unfilled resin systems. The filled resin contains particles that reduce the CTE in the z-direction, and according to the results in this study, they also inhibit crack propagation and failure look. Compared to the number of loading cycles until the failure occurs, the filled resin is better, but the average pull strength is higher for those unfilled. In a filled system, the glass cloth is not visible after cratering because the crack does not tend to propagate deep into the material. The same study further deals with the impact of the pull rate, pull angle, amount of reflow cycles, and degradation mechanisms on pad cratering. Godbole et al. [19] investigated the connection between the pad cratering and the pad placement within the PCB together with the effect of reflow cycles and moisture exposure. For pad cratering determination, three methods have been established, and they are described in the IPC-9708 standard. These methods are ball shear, ball pull, and pin pull testing depicted in Figure 1. They are presented in more detail in [20], including the description of their benefits and drawbacks. Cia et al. [21] took advantage of the pin pull method to evaluate pad cratering after multiple reflows and accelerated thermal cycling. Susceptibility to pad cratering is influenced by the reflow profile peak temperature, and thermal aging plays an important role if the temperature is above Tg [22]. The possibility of pad cratering is enhanced by inducing stress during the release of latent heat when the solder solidifies, as Dušek and Rudajevová mentioned in their study [23]. Latent heat locally raises the temperature under the pad and keeps the resin in a viscoelastic state, while the surrounding PCB is under Tg and is already rigid.

Copper pads intended for soldering tend to be covered by the combination of copper oxides [24]. Surface finish utilization is practically unavoidable for preventing the reaction of copper with oxygen in the air. Moreover, its utilization is supported by the fact that current soldering pastes containing no-clean fluxes with low activity. Oxidized surface causes insufficient solderability. The solder wetting without using very aggressive flux is impossible in the case of exceeding oxide thickness threshold [25]. Surface finish influences the reliability of solder joints [26,27,28]. Therefore, the selection must comply with the following device operation. Surface finishes are deposited in various ways according to the requirements of the material, ensuring protection.

Two surface finishes, HASL (hot air solder leveling) and OSP (organic surface preservative), are presented in this study. Hot air solder leveling finish involves soaking the PCB into the molten alloy. In an area of lead-free soldering, the PCB is exposed to 250 °C. In addition, after taking out of the bath, the PCB undergoes a hot air knife in order to remove excess solder and make the thickness more uniform [29]. An organic solder preservative is deposited by immersing the PCB into liquid, or in horizontal conveyorized processing, the board is sprayed. The properly cleaned PCB and micro-etched copper pads are exposed to liquid consisting of an organic compound. The organic component is dissolved first in water and organic acid. Then the PCB with OSP coating is left to dry under conditions not exceeding 50 °C. The whole process is quite simple, and it does not affect the PCB in terms of thermal shock, unlike HASL. The compatibility of OSP with lead-free soldering is being solved by developing new substances, providing higher heat stability [30]. OSP coating is cheaper and more planar than HASL. On the contrary, HASL is more resistant to mechanical damages, moisture, and temperature and reduces storage demands.

A distinction between OSP and HASL in the field of soldering and PCBs was determined by several studies. Dušek et al. [31] detected stronger mechanical resistance of soldered joints for HASL. In the work of Vasko et al. [32], HASL provided better wettability than OSP. The results showed stronger joints made on pads covered by HASL, too. Reliability tests done by Zhou et al. [33] proved comparable results after thermal cycling for both finishes and better endurance for OSP in the drop tests.

Within PCB testing, another strength of the interface is studied. Peel strength is commonly performed to assess the bond between the resin and pressed conductive foil. Peel strength is mainly given by the foil roughness [34,35]. Interesting research in terms of our study was conducted by Liu et al. [36], who investigated the peel strength after thermal shock inflicted on the copper-clad laminate. The study showed the negative repercussions of the thermal shock on the adhesion of copper patterns to the substrate.

Our investigation focused on the evaluation of the thin resin layer adhesion beneath the copper pattern to the reinforcement. The strength of this adhesion (in the article called bond strength) is a crucial indicator for the formation of presented failure—pad cratering. As it was mentioned, there are no possibilities for repair. Therefore, there must be a wide range of evaluating studies dealing with this failure. Then, during the PCB’s design and material selection, the experience gained by the studies helps to avoid or decrease the risk of tearing the pad out of the substrate. Resins used for the production of the laminated composites have various thermal properties, and it is expected to have a different response to thermal loads. To cover this concern, three laminates characterized by diverse Tg values were chosen. Even though the two variants of epoxy resin were subjected to similar evaluations, a comparison with polyimide resin and in relation to other testing parameters has not been performed. Another concern appears as the resins do not have the same adhesion to reinforcement. In addition, the bond might change after thermal or mechanical stress. The bond strength of the soldering pad to the substrate has not been deeply evaluated considering the surface finish. Further, an indicated issue was tested directly under an elevated temperature, which gives importance to this study. A new method was developed for the purposes of strength testing, which reduces the problems related to failure mode recognition. The used method ensures detachment at the interface of the resin and filler.

According to one method of surface finish application, the consequences left on the laminate caused by this part of PCB fabrication should not be neglected. Adverse effects in the form of deviations in thermal expansion, drops in Tg value, or changes in material reaction thermomechanical loading ought to be checked. Changes in material behavior can cause some of the negative difficulties described in the paragraphs above in the course of soldering or consequent device operation. This relevant issue is not addressed in previous studies. Hence, it was observed in more detail within this study.

## 2. Materials and Methods

**Figure 2:**
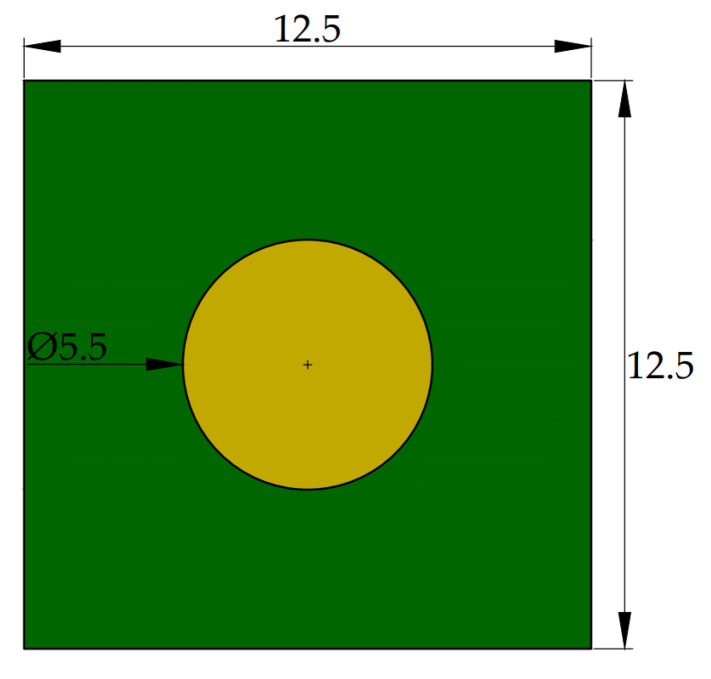
Design of the testing board.

Three types of laminates were selected based on their Tg value. A set of samples included the basic variant of DICY-cured epoxy FR4 laminate (Tg1), which is still widely utilized in consumer electronics. Phenolic-cured epoxy FR4 (Tg2) and polyimide G30 (Tg3) resins in combination with glass cloth were further laminates involved in the evaluation. This laminate was intended for high-temperature usage to ensure the required reliability. The list of used materials is shown in Table 1. The testing boards made of listed laminates were designed to contain the spacious circular soldering pad with a diameter of 5.5 mm. The overall size (12.5 × 12.5 mm^2^) of one specimen (see Figure 2) was adjusted to fit the size available in the tool. For each sample version, 15 pieces were assessed.

**Figure 3:**
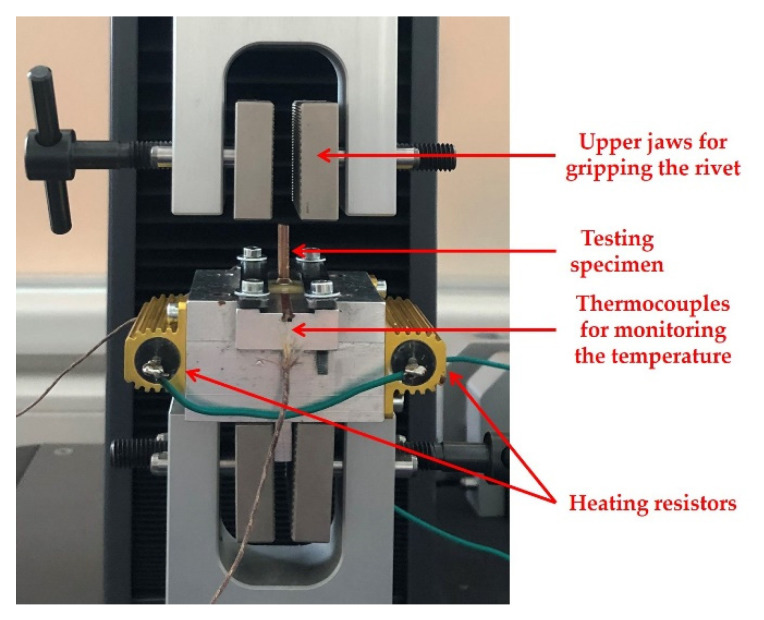
Sample fixed in the tool.

The tool for placing the sample as a part of the employed tensile testing machine X250-3 (Testometric, Rochdale, Great Britain) is visible in Figure 3. As the previous section suggests, HASL (H) and OSP (O) were chosen to assess if some finishes can make the bond between the glass cloth and the thin layer of the resin under the soldering pad weaker, thereby contributing to the pad cratering phenomenon.

A novel approach for analysis was established to eliminate a majority of the aspects influencing the strength of the bond between the resin and the glass cloth during the mechanical test. The newly utilized method allows for focusing on the desired bond and thereby material and technology comparisons. There are more possibilities of resin and reinforcement suitable for substrate production and many other types of material inputs (e.g., surface finish and solder mask) involved in PCB fabrication. These materials must be applied on PCBs by a technological process that may be incompatible with the selected base materials and erode the original properties. Further, this method can be adopted for various mass soldering techniques used for surface mount technology (e.g., hot air, vapor phase, or infrared soldering). It should be noted that utilizing soldering pads with a relatively large area does not influence the mutual comparison of technology and material combinations.

**Figure 4:**
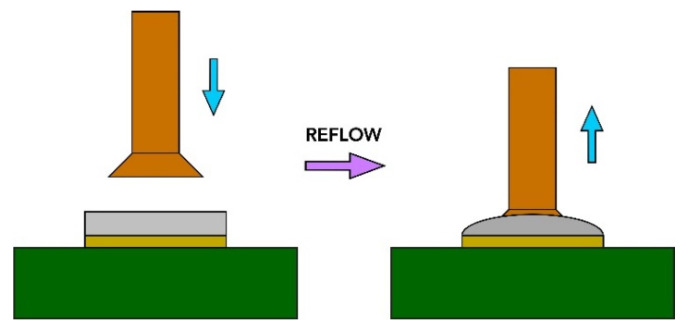
Testing method—rivet mounting.

**Figure 5:**
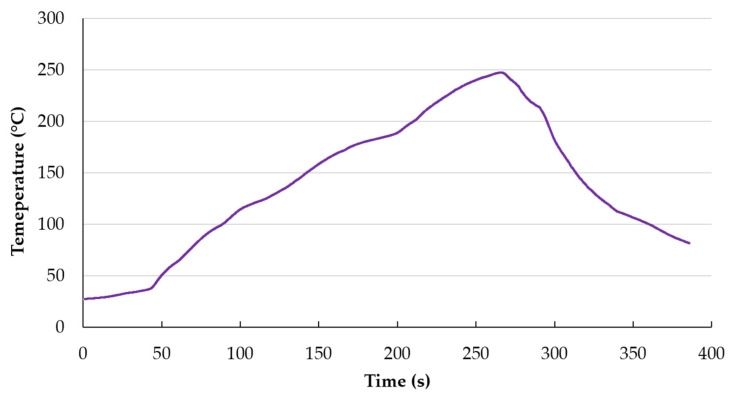
Temperature profile achieved for reflow in the oven Mistral 260.

A copper countersunk head rivet was mounted to transfer the tensile force to the soldering pad (see Figure 4). Rivets were mounted to the soldering pads using the conventional reflow method. At first, lead-free soldering paste SAC305 (Sn-3%Ag–0.5%Cu) was applied on the soldering pads using stencil printing. After manual assembling of the rivets, the PCBs were reflowed in a forced air convection oven Mistral 260 (Technoprint, Ermelo, The Netherlands) with three temperature zones. The temperatures of the zones were adjusted to create the temperature profile suitable for the chosen lead-free solder paste (see Figure 5). Before every measuring test, the sample was attached to the tool in that way to allow the pad to detach from the substrate freely. A rivet with a 3 mm diameter was firmly fastened into the upper jaws of the deformation device before the test. The speed of the upper jaw was set at 1 mm/min.

Tests were performed at ambient temperature (AT) and an elevated temperature (ET) of 100 °C. The preheating of samples for purposes of testing at an elevated temperature took place in an oven UN55 (Memmert, Schwabach, Germany). Preheating was used to achieve a more uniform temperature at the samples. The preheating process lasted for approximately 30 min. After, the sample was placed and fastened into the heated tool. The heat of the tool was supplied by energy dissipation in two resistors connected in a series to the voltage source. The temperature in the tool and on the sample was monitored by thermocouples (type K) located in the tool directly under the sample.

Measurements to determine the Tg and possible influence of the surface finish on the behavior under thermal action were conducted on TMA Q400EM (TA Instruments, New Castle, DE, USA) equipment. This apparatus served for both the TMA and DMA procedures. During thermomechanical analysis, square-shaped samples with an edge length of 8 mm and thickness of 1.5 mm were placed on the stage and heated to a temperature that was approximately 40 °C above the transition temperature given by the datasheet. The heating ramp was established at 5 °C/min. A nitrogen atmosphere was available in the heating chamber. The Tg estimation was performed as Yong et al. [37] proposed. Accordingly, analysis of the curve (temperature dependence of dimensional change) was obtained by a sensitive probe while having plotted the first derivation of that dependency. The first derivative of dimension change with respect to temperature helped to define the onset and the end of glass transition. The sharpest dimension change identifies the onset, thereby the lower temperature boundary. The peak or stabilization of the first derivation points to the upper limit. The temperature closest to the midpoint of the stated temperature interval was taken as the Tg.

The DMA measurements were performed using an aluminum fixture with supporting rollers that formed a three-point bending system in conjunction with a flexural probe. The rollers on which the samples were put were at a 10.16 mm distance from each other. For DMA purposes, the delivered composite substrates with a thickness of 1.5 mm were cut to obtain rectangular-shaped specimens with a width of 3 mm and length of 15 mm. Temperature conditions during DMA were similar to the TMA measurements. The only maximal temperature was adjusted to capture complete significant transitions of monitored quantities. Modulated force amplitude was set to ±0.2 N that was applied to the sample at a frequency of 1 Hz. The static force was 0.25 N. Each type of sample was analyzed three times. One exemplary diagram is comprised in the results.

**Figure 6:**
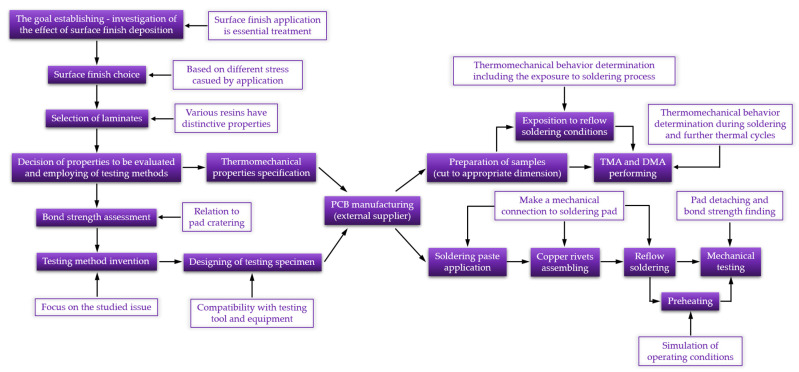
Research methodology.

The methodology of the study is presented in Figure 6. The procedure diagram items are labeled to describe the motivation of the work steps. Details of several of the motivations are explained in Table 2 which support the diagram.

## 3. Results

### 3.1. Bond Strength Evaluation

**Figure 7:**
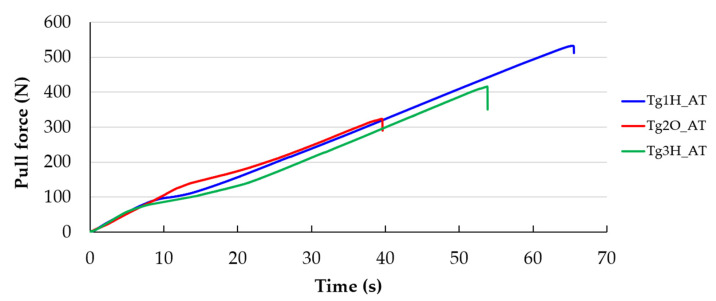
Dependency of pull force on the time.

The whole pulling process was recorded, and the maximum force for further evaluation was consequently determined from the sampled data. The course of force allows for definite detection of the highest force. Examples of pulling course are depicted in Figure 7. This course was typical for every sample. The detachment of the pad was accomplished at one moment without any gradual tearing off.

**Figure 8:**
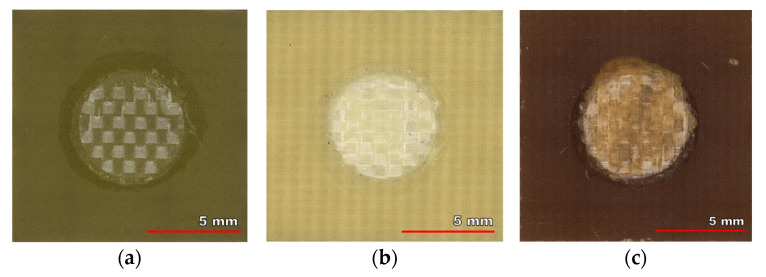
Exposed glass cloth after tearing the pad: (**a**) FR4 low Tg; (**b**) FR4 high Tg; (**c**) G30.

The failure mode of the pulling test for each laminate is visible in the photo of tested samples shown in Figure 8. The pads are completely torn from the substrate, and the glass cloth is visible. An advantage of the used method was confirmed, because no part of a specimen was destroyed in another area than was required. Thus, any destruction at the interfaces of solder and pad, rivet and solder, nor breaking the rivet did not happen. Obviously, thanks to its design and larger cross-sectional dimensions.

**Figure 9:**
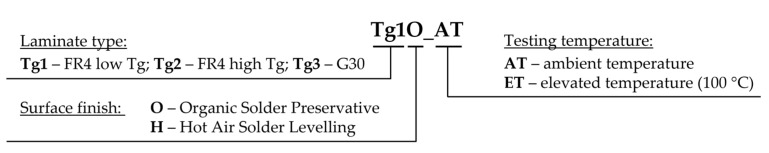
Explanation of the sample marking.

The sample marking is explained in Figure 9. It corresponds with the bond strength’s evaluation. The beginning of the marking without the abbreviation that follows the underscore was used in the whole text.

**Figure 10:**
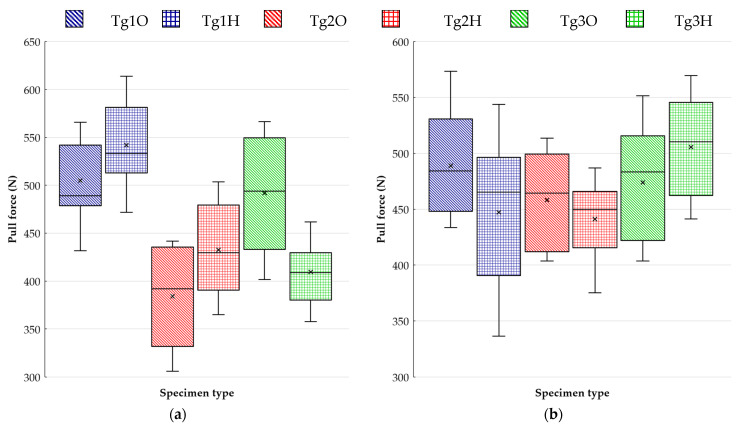
Pulling test results: (**a**) at ambient temperature; (**b**) at elevated temperature (100 °C).

The pulling test results summarized in the boxplots (see Figure 10) and in Table 3 show the strong dependency of the bond strength on the resin type. Testing at ambient temperature, as reported in Figure 10a, points to a slight impact of surface finish on the studied issue for both versions of FR4 laminates. Despite the presumptions, pads with an HASL surface finish and the resin under them did not lose the rate of adhesion to glass reinforcement. Our tests showed a certain improvement in adhesion that could result from the softening of the resin during the surface finish application, because these resins have a Tg far below the temperature of solder bath. The thermal treatment provides the possibility for the resin to adhere better to the reinforcement.

The ability of the surface finish to influence the bond between the resin and glass cloth was detected to a greater extent for the laminate with the highest Tg. It was found that the resin where soldering pads had an HASL surface finish had a smaller bond strength by 3.2 N/mm^2^ on average than those with OSP.

As to testing at an elevated temperature, it can be stated that the distinctions in bond strength among the used laminates diminished. The effect of the surface finish persisted, but the test proved worse resin cohesion to glass cloth for HASL in the case of FR4 laminates. In particular, FR4 with a lower Tg indicated a noticeable difference. In contrast to the testing at 25 °C, the G30 substrate with OSP became prone to pad cratering than pads covered by an HASL surface finish, but the difference in average force values was not so evident.

**Figure 11:**
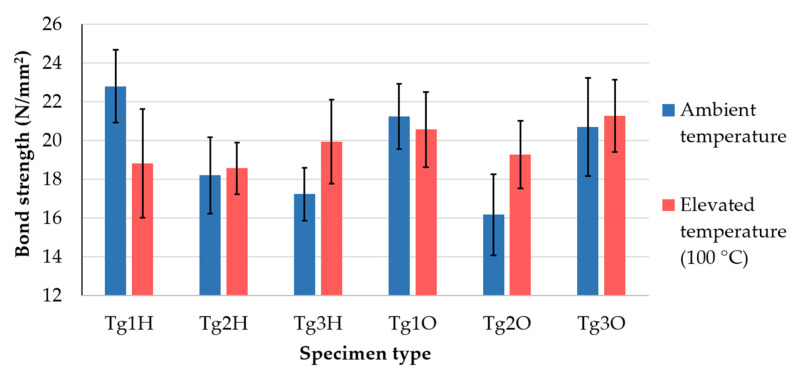
Comparison of the results according to the testing temperature.

The bar chart in Figure 11 offers a clearer comparison of average bond strength values between testing at room (blue bars) and elevated temperature (red bars). These values were calculated by dividing the mean value by the surface of the soldering pad. Error bars were derived from standard deviation. The higher testing temperature significantly affected the bond strength in specimens made of low-Tg FR4, G30 with HASL, and high-Tg FR4 covered by OSP. It was confirmed that the decrease in bond strength during testing at 100 °C for the least thermally resistant material, but Tg1O was affected very little. Laminates for thermal application behaved the opposite way when all the specimens showed higher strength even though the differences were almost negligible for Tg1O, Tg2H, and Tg3O. The PCB supplier declared the manufacturing (temperature and pressure during copper cladding) in accordance with the recommendations of the laminate producer. In connection with this fact, another question arose as to whether the optimizing procedure parameters of the pressing process may reveal whether the studied strength of adhesion can be improved.

The lower bond strength attained for the phenolic-cured substrate was consistent with the results obtained by Ahmad et al. [38]. Their study dealt with pull strength associated with pad cratering contained DICY and phenolic-cured laminates, too. Tests within the study revealed an approximately 50% smaller pull strength for the phenolic-cured material. The results relating to epoxy resins and surface finish may be roughly compared with [39], which dealt with a similar issue. Interlaminar strength in the epoxy resin and glass fiber system was assessed but differed in thermal shock caused not by fast heating but rapid cooling down of the heated samples. Nevertheless, the insignificant influence of this thermal load was likewise discovered.

The outcome of testing at an elevated temperature can be compared with the research conducted by Roggeman and Jones [40]. Laminates based on epoxy resin reported a moderate drop of pad strength when tested at 65 °C. Here, a discrepancy with our results obtained for high-Tg epoxy laminate might be registered, because we measured higher values when testing at 100 °C.

### 3.2. Analysis of Thermomechanical Properties

Observation of material mechanical properties during the heating cycle was the second part of this paper. It must be noted that at first, all thermomechanical analyses were conducted using the substrate that had not passed the reflow soldering or had not been exposed to higher temperatures after delivering the produced PCB samples. After performing these analyses and based on the results, both the TMA and DMA of the Tg1 specimens were carried out after exposure to the reflow profile shown in Figure 5. Each sample was analyzed in three cycles, but sometimes, only two cycles are included in graphs when the third cycle did not differ significantly from the second one.

**Figure 12:**
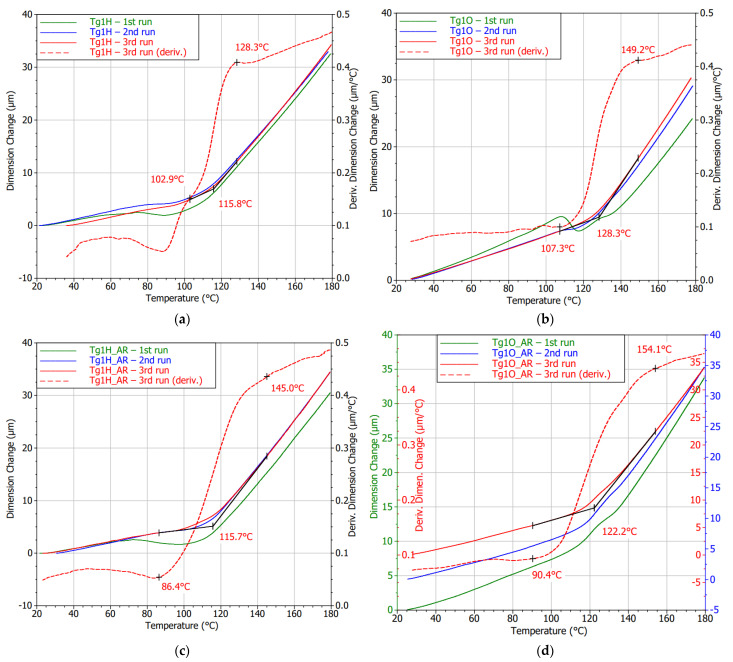
TMA diagrams of the Tg1 substrates and with surface finish: (**a**) HASL; (**b**) OSP; (**c**) HASL after reflow (AR); (**d**) OSP after reflow (AR).

The curves that are depicted in Figure 12 were obtained for the FR4 substrate with lower Tg. Shape undulation of laminate with an OSP finish is observable in the glass transition surrounding, especially during the first heating cycle. The gradual curing process and stress release of the laminate were detected by the following measurements of the same sample. This observation is in accordance with the findings in the literature conducted by Rudajevová and Dušek [41]. A new outcome can be found for the impact that causes the application of an HASL surface finish from a thermomechanical point of view. The thermal shock caused by dipping the board into the molten solder had an adverse effect on the non-fully cured substrate. Even though a specific curing process can be observed for Tg1H, it did not lead to possible improvements in the Tg value, and the magnitude remained diminished. A method of Tg derivation described previously was observable in the TMA figures, and Tg values derived from those curves are summed up in Table 4. The values in the table are stated as an average of the given measurement cycles (second or third).

Samples that underwent the reflow process showed this treatment to serve for their curing. It can especially be seen in Figure 12d. A throb of the first cycle curve was not so enormous as it is in Figure 12b. The curves must have shifted in the *y*-axis; hence, three *y*-axes were utilized to make them visible and compare their shapes. It can be said that the changes in the shape by performing more cycles were negligible. Using TMA in the case of samples that had been subjected to HASL application and consequent reflow cycle led to the detection of an irreversible decrease in Tg.

**Figure 13:**
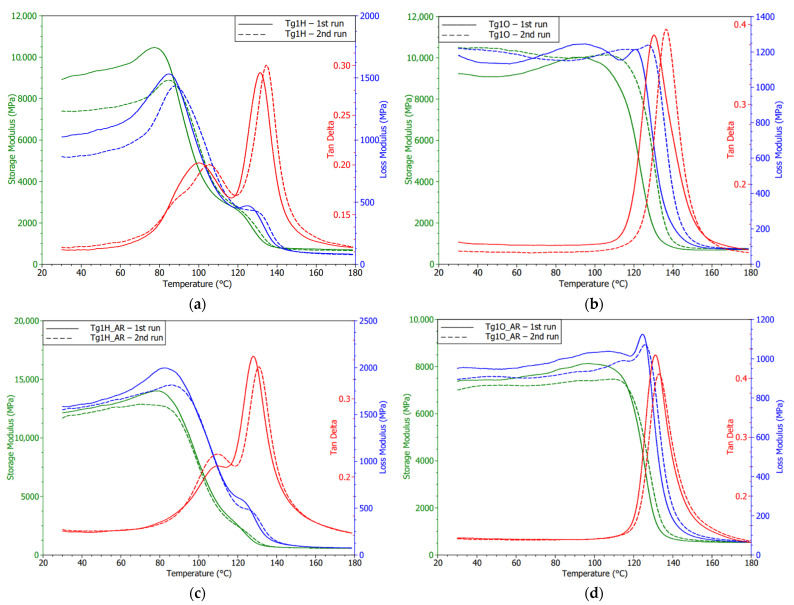
DMA diagrams of the Tg1 substrates and with surface finish: (**a**) HASL; (**b**) OSP; (**c**) HASL_AR; (**d**) OSP_AR.

Detections obtained by TMA were confirmed by conducting DMA measurements. Considering the monitored dynamic properties of Tg1O, subsequent curing of the resin was noticed. It resulted in the delay of the storage modulus decrease during the second cycle. Storage modulus fall takes place at a higher temperature, while Tg given by the loss modulus peaks remained unchanged. The results shown in Figure 13a verify the premature softening of the Tg1H samples at approximately 85 °C during the first heating. In the second run, a slight shift of the softening point at approximately 5 °C was observed. In addition, the decline in the point of storage modulus was related to the ability of the material to withstand mechanical stress without deformation. The residual of the proposed Tg can be recognized in recorded storage or loss modulus. It may indicate that the degradation of the polymer resin had not influenced the whole volume of the substrate. The board was dipped in the solder bath for few seconds; therefore, the warm-up was not passed into the resin located further away from the surface.

Disruption of cross-linking is a probable reason for the detected dissimilar and wavy peaks of loss modulus. Although Margem et al. [42] tested epoxy matrix using different curing agents, insufficient cross-linking rates lead to the occurrence of the same phenomena in DMA results. Polanský et al. [9] studied the effect of thermal influence with a longer duration and achieved double peaks explained by degradation of inner structure, changes in material surface profile, and the appearance of delamination.

Diagrams of DMA (eventually TMA measurements) for samples that went through the soldering oven evidently testify that a slow increase in temperature to 250 °C did not have a lasting impact on the thermomechanical properties. Laminate with OSP did not report any breaking changes in material behavior, only some slight post-curing consequences influencing the laminate were observed. Mostly, the second heating cycles of both finishes were connected with the lower absolute values of storage modulus in the glassy area. This means the lower stiffness of the laminate is associated with the higher energy stored by the system. Another reason for the varying storage modulus was the strength of the adhesion between the fiber and the matrix within the composite as Keusch and Haessler [43] researched in their work.

**Figure 14:**
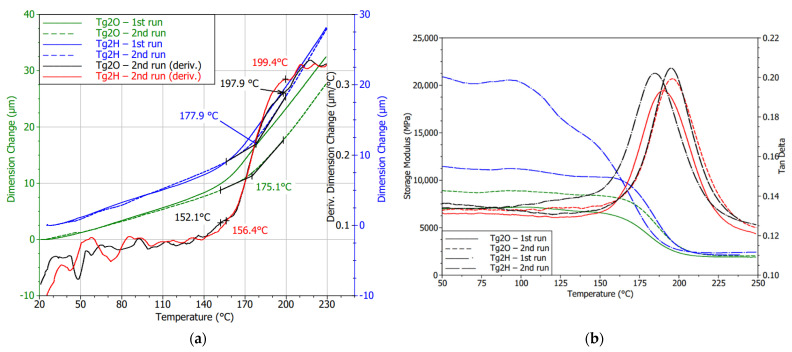
Diagrams of the Tg2 substrate including both surface finishes: (**a**) TMA; (**b**) DMA.

The effect of HASL application on the Tg2H (high Tg FR4 substrate) was demonstrated because it reduces internal stress and improves the curing rate (see Figure 14a). Deviation in the Tg region was less noticeable, and the curve of the first measurement was closer to the course achieved during the second heating run. A fall in Tg by 2 °C was found, but it was not statistically significant as in the case of Tg1H (low Tg FR4 substrate). On the other hand, DMA measurement brought the ascertainment that the laminate temporarily reported a higher storage modulus, and then its decline followed as is visible in Figure 14b. However, by heating the sample, this property disappeared. In the second cycle, the storage modulus met a typical shape comparable to the substrate with soldering pads covered by OSP, but the storage modulus remained high. The change in interface bonding can explain this finding. The storage modulus of the second analysis testified that there was no impact on the Tg value, too. The bend in the storage modulus (second cycles) suggests a Tg value of approximately 175 °C, which supports the TMA results.

**Figure 15:**
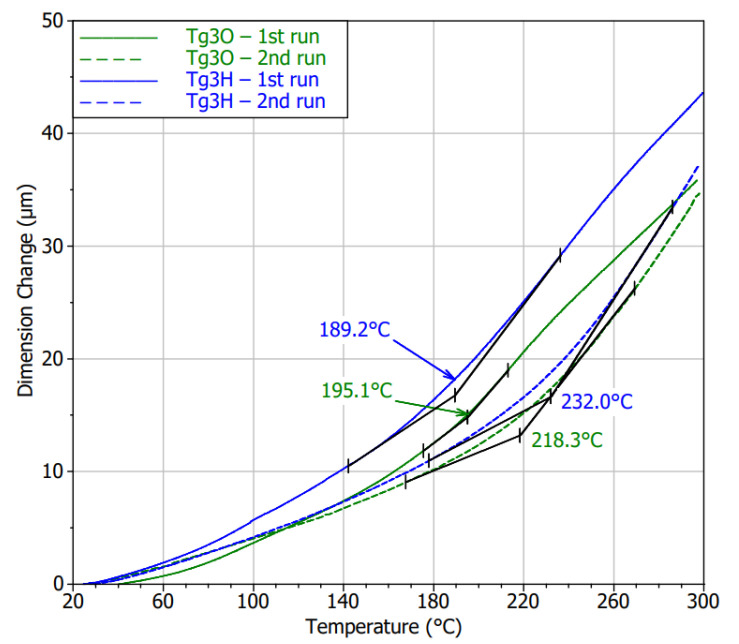
Comparison of the results according to the testing temperature.

TMA analysis revealed distinctive expansion behavior in the first heating cycle compared to the second one. Except for the undulation of the curves during the first cycles, particularly the HASL specimens exhibited higher CTEs in the glassy state. The thermomechanical performance of both specimen types became nearly the same in the second measurement run. Further, the curve bend was not as striking as it was typical for the previous laminates. Hence, inaccuracy in Tg determination may occur. Regardless of this fact, the Tg was almost independent on the surface finish, and the values increased by undergoing the thermal treatment under the conditions of the TMA measurements as can be seen in Figure 15. The Tg for Tg3H was detected to be higher by 3.5% on average, which can be found to be relatively insignificant. However, noticeably lower values than those declared in the datasheet must be considered for a potential application.

**Figure 16:**
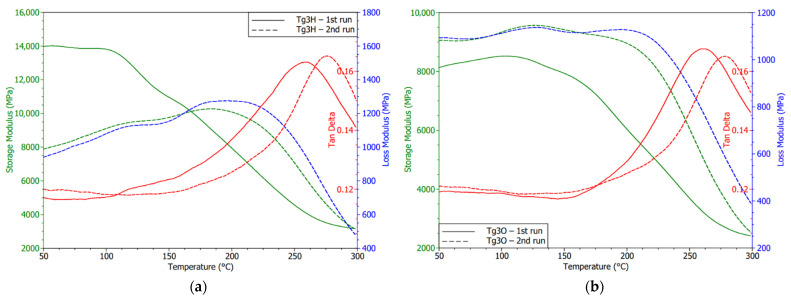
DMA diagrams of the Tg3 substrate with the following surface finish: (**a**) HASL; (**b**) OSP.

Both G30 laminates using HASL and OSP showed changes in the storage modulus (see Figure 16), thus the mechanical properties comparing the first and second cycles. An increase in the storage modulus and extension of its glassy state in the OSP specimen may denote an improvement in the interface between the filler and the reinforcement. A low diminishing of the peak magnitude of tan δ correlated with this statement. Generally, it was found that the Tg3 laminate had the lowest tan δ magnitudes, which means smaller energy dissipation options. Storage modulus curves or optionally the peaks of the loss modulus suggest the Tg values were under 250 °C as the TMA results also indicated.

## 4. Conclusions

We evaluated the strength of the bond between the glass cloth and selected resins. A new testing approach was used considering reflow soldering. Our results revealed the dependence of adhesion on the resin type. Furthermore, the role of surface finish on bond strength was demonstrated. However, the bond strengthening or weakening rate depended on the resin. The same material combinations were tested at an elevated temperature of 100 °C. It was proved that the resin with the lowest Tg tended to lose adhesion to glass cloth. The bond strength in other assessed laminates did not show a higher tendency to become weaker when tested at 100 °C. Actually, in all cases, a higher force necessary for detaching the testing pad was measured. Simultaneously, the differences among the sample types were significantly smaller for testing at elevated temperatures. The best resistance to tearing the pad from the substrate was detected for the sample marked as Tg1H—laminate containing the resin with the glass transition temperature of 135 °C (according to the datasheet) and HASL finish. The evaluation at 100 °C revealed the strongest bond for the sample marked as Tg3O—G30 laminate on which the pads were protected by OSP.

The second part of the study utilized a thermomechanical analyzer to establish material behavior changes in consideration of the surface finish. The TMA and DMA results were included. In the matter of used surface finish, conventional substrate (marked as Tg1) was highly impacted by HASL application resulting in permanent degradation of the laminate. Both methods also indicated the decline of Tg. Specifically, the Tg shift to a lower magnitude derived by TMA was almost 10 °C. A direct effect of thermal shock caused by the application of HASL finish was confirmed. As the testing of the same laminates after simulating the reflow process (i.e., the gradual heating of the laminate to temperature reaching 250 °C and subsequent measuring of the laminate with OSP surface finish) showed observable Tg movement to a lower magnitude but not as significant as the difference between sample Tg1O (with OPS surface finish) and Tg1H (with HASL surface finish) before reflow.

Composites with higher temperature resistance did not undergo permanent deviations in the thermomechanical performance regarding the border between the glassy and rubbery region. The post-curing internal process leading to stabilization of the material in the vicinity of Tg as well as stress relief were detected for these laminated composites, too. Moreover, laminates covered by HASL tended to a transient increase in stiffness. That effect vanished, because second cycles reported common diagrams of storage modulus.

## Figures and Tables

**Table 1:** Laminates used for the investigation.

Producer	Type	Grade	Glass Transition Temperature	Samples Marking
JIANGSU RODA ELECTRON MATERIAL, Rudong, China	RD140	FR4	135 °C	Tg1
TECHNOLAM, Troisdorf, Germany	NP-175F	FR4	170 °C	Tg2
Göttle Leiterplattentechnik, Königsbrunn, Germany	VT-901	G30	250 °C	Tg3

**Table 2:** Explanation of the main motivations.

Operation	Motivation
Investigation of the effect of surface finish	The impact of the technological process (surface finish application) on the laminate evaluation is crucial.
Surface finish choice	Two surface finishes were chosen regarding thermal circumstances during application. HASL application is accompanied by thermal stress, whereas OSP is not.
Selection of laminates	Various resins or their modifications have different thermal properties and adhesion to filler.
Bond strength assessment	The strength of the adhesion of the soldering pad, specifically resin to filler, is significant in relation to failure–pad cratering occurring on the PCBs.
Reflow soldering	Except establishing the mechanical connection, the bond strength results respect the effect of this treatment, which is an essential step in electronic assembly.
Preheating	Specimens tested at an elevated temperature were preheated to achieve an equal temperature throughout the sample. Consequent mechanical tests performed at 100 °C were realized in order to simulate field conditions.
Exposition to reflow soldering conditions	It was included to verify the effect of surface application, i.e., comparison of slow and rapid heating.
Thermomechanical analysis (TMA) and dynamic mechanical analysis (DMA)	Observation of material behavior in the surrounding of Tg and detection of Tg value displacement. Assessment of material response during mechanical loading in conjunction with temperature rise. More measurements cycles were conducted to determine the response during soldering and, consequently, the effect of the thermal loading.

**Table 3:** Summary of the measured and calculated values.

	Tg1H_AT	Tg2H_AT	Tg3H_AT	Tg1O_AT	Tg2O_AT	Tg3O_AT	Tg1H_ET	Tg2H_ET	Tg3H_ET	Tg1O_ET	Tg2O_ET	Tg3O_ET
Mean (N)	542.0	432.5	409.6	505.0	384.2	491.9	447.2	441.2	473.9	488.8	458.2	505.6
Minimum (N)	471.7	365.1	357.8	431.5	305.9	401.5	336.5	375.1	403.6	433.5	403.7	441.3
Maximum (N)	614.0	503.5	461.8	565.8	441.8	566.5	543.9	486.9	551.5	573.6	513.7	569.5
SD (N)	44.4	47.1	32.6	39.9	49.7	60.0	66.7	31.6	51.4	46.3	41.4	44.6
Bond strength (N/mm^2^)	22.8	18.2	17.2	21.3	16.2	20.7	18.8	18.6	20.0	20.6	19.3	21.3

**Table 4:** Glass transition temperatures (expressed in Celsius) determined by TMA.

Tg1		Tg1_After Reflow		Tg2		Tg3	
HASL	OSP	HASL	OSP	HASL	OSP	HASL	OSP
116.2	127.9	115.5	123.4	177.1	176.7	228.8	221.4

## Data Availability

The data presented in this study are available on request from the corresponding author.
